# Conversations About Congenital Clubfoot: Investigating How Parents Share Information About a Structural Diagnosis With Their Children

**DOI:** 10.7759/cureus.48576

**Published:** 2023-11-09

**Authors:** Danika Baskar, Maxance Ngnepieba, Pooja Paul, Nicole A Segovia, Steve Frick

**Affiliations:** 1 Orthopaedic Surgery, Stanford University School of Medicine, Stanford, USA; 2 Developmental Psychology, Stanford University, Stanford, USA

**Keywords:** support, education, information sharing, family, parent, guidance, talipes equinovarus, clubfoot

## Abstract

Background and objective

Clubfoot is a common congenital musculoskeletal condition that is treated with manipulation and casting in the first few weeks of life, followed by bracing that extends into early childhood. While children typically do not recall treatment with Ponseti casting in infancy, childhood treatment and monitoring may result in a sense of heightened awareness. In light of this, this study explores how parents share information about clubfoot diagnosis and guide their children in understanding the importance of treatment.

Methods

Parents of clubfoot children aged 5-18 years were eligible to participate. Primary recruitment was done through social media via Facebook clubfoot support groups. Participants who gave consent completed an electronic survey and were invited to take part in a semi-structured interview to share additional experiences. Significant themes elicited from study interviews were analyzed along with survey responses.

Results

Survey responses were received from 74 parents, and 23 participated in the semi-structured interview. Of note, 91% of parents indicated discussing clubfoot with their children, beginning at a median age of three years. The age at which parents first discussed clubfoot with their child was significantly earlier for those who “strongly agree” that their children understand their condition versus those who “agree". Although 68% of parents indicated that receiving guidance from their orthopedic provider would be helpful for these discussions, only 18% noted receiving direct advice. Recurrent themes across interviews included being open and honest about the children's diagnosis and treatment, aiding the children in taking ownership of their diagnosis, and validating emotional responses throughout treatment.

Conclusions

This study provides valuable insights into initiating conversations with children about structural diagnoses like congenital clubfoot. Recurrent themes from conversations with families provide information on helpful strategies to encourage early discussions about clubfoot diagnosis and treatment to aid children in taking ownership of their diagnosis.

## Introduction

Congenital clubfoot or talipes equinovarus is a complex structural deformity of the foot and leg that develops early in a child’s gestational development [1,2]. With an incidence of one in 1000 live births, clubfoot is one of the most common congenital musculoskeletal conditions that requires early treatment, typically beginning in the first few weeks of life [2,3]. Treatment for clubfoot historically involved invasive surgical release of the soft tissues and joints to reposition the bones of the foot. In the late 1990s, Dr. Ignacio Ponseti’s method of serial manipulations and casting was championed by parents seeking nonoperative treatment that would provide improved clinical outcomes [4]. Since then, the Ponseti method, which consists of weekly manipulations and casting, and in most cases an Achilles tenotomy, has revolutionized the initial treatment of clubfoot as it is informed by a deep understanding of the functional anatomy that underlies the pathology [4].

Clubfoot treatment is unique in that it requires a significant commitment from the patient’s family to ensure successful outcomes. After the initial correction of deformity with the Ponseti method, children are fitted with a foot abduction brace to be worn full-time (23 hours per day) for the first three months after casting, and then at night and naptime (12-14 hours per day) every day until the age of two to four years. Possible extension of treatment duration may be necessary depending on the severity and etiology of the child’s clubfoot. The development of relapse later in childhood also presents challenges to treatment as it may involve repeat Ponseti casting and surgical correction in some cases [2,5]. Children with congenital clubfoot typically do not recall the initial early treatment in infancy. Aspects of treatment as the child ages, such as daily bracing, possible repeat casting and surgery for relapse, physical therapy, and regular appointments with their healthcare providers, can lead children to develop a heightened sense of awareness and curiosity. Children with siblings and those attending school or daycare may also begin to independently ask questions about their affected foot and treatment as they notice differences in function and appearance when compared to their peers. While this opens opportunities for parents to engage in discussions to help educate their child about their clubfoot diagnosis and treatment, navigating these conversations can be difficult given that their child is asking about a congenital disorder.

The concept of health ownership has been defined as taking control, accepting responsibility, and being accountable for one’s health [6]. Achieving health ownership involves having the necessary knowledge about one’s condition to implement and sustain behaviors that positively impact outcomes. Patient education and empowerment from providers and caregivers form vital components of this process [6,7]. For young children, parental guidance and education through early conversations create opportunities to support them as they learn about their condition. Literature investigating conversations between caregivers and their children on disclosing information about a child’s congenital diagnosis is extremely limited, with few existing studies focused on discussions surrounding autism spectrum disorder and pediatric cancer [8-10]. A survey study looking at parental experiences about discussing autism spectrum disorder with their child highlights the importance of having open and honest conversations that occur gradually to help guide the child in taking ownership of their diagnosis. This study additionally mentions how tailoring discussions to the specific needs of the affected child and professional support for parents may be beneficial in preparing caregivers for these conversations [8]. Also, the complex interplay between overloading medical information, the emotional impact of shared knowledge, and the lack of experience with having related discussions among families must also be borne in mind as this introduces additional levels for consideration [9,10].

Sharing information with the intention of educating children about their diagnosis while trying to avoid making them feel different or creating the impression that something is wrong can be especially challenging for parents. To date, no studies have examined similar discussions among families with children who have congenital structural anomalies and physical conditions. While there has been a recent uptick in research interest surrounding several aspects of congenital clubfoot outcomes and treatment, conversations between parents and their children about clubfoot diagnosis have yet to be investigated thoroughly. The purpose of this study was to explore discussions among families with clubfoot children to learn how parents share information about clubfoot, answer related questions, and guide their children in understanding their diagnosis and the importance of treatment. Information gathered from study surveys and parent interviews may provide insights for families who have similar conversations about physical diagnoses like congenital clubfoot with their children in the future.

## Materials and methods

Study design and setting

We employed a prospective cohort study design. Parents of children diagnosed with congenital clubfoot who were between the ages of 5-18 years and had received prior treatment or were currently undergoing treatment at the time of enrollment were eligible to participate in the study. Primary recruitment was done through social media via Facebook groups bringing parents and families together for support surrounding clubfoot-related topics. An infographic about the study was provided to Facebook group administrators to share with other members of the group. Parents interested in the study were directed to an electronic screening survey to confirm their eligibility to participate. Informed consent was obtained electronically, after which participants could complete the study questionnaire. Additional study recruitment was performed through division faculty practices where eligible participants were provided a study infographic at the end of their scheduled appointment. In addition to participation in the study survey, parents were invited to take part in an optional semi-structured interview with the research team to elaborate on their responses from the survey and openly share significant experiences. Interviews with parents were conducted virtually over the institution’s Zoom platform, with audio and video recording to aid in the transcription of responses. All study activities underwent thorough review by the Stanford University School of Medicine Institutional Review Board (IRB) at the primary institution under protocol #60669 where this research was conducted and received approval for the methods described.

Questionnaire design and data analysis

The study survey and semi-structured interview were developed by a multidisciplinary team of researchers by drawing on their experience in pediatric orthopedics, medical anthropology, and developmental psychology. The questions were designed to learn about information exchange between parents and their children concerning clubfoot diagnosis, challenges related to having these conversations, and strategies to help their child understand their condition. Additionally, a subset of questions was included to understand the impact that living with clubfoot had on the child from the parent's perspective. A pilot group of clubfoot families was asked to provide feedback on the survey and interview questions, leading to modifications in the final questionnaire. After data collection was complete, quantitative and descriptive analyses of survey responses were performed. Simple linear regression models were run using R Studio version 1.1.456 and a two-sided level of significance of 0.05. Semi-structured interview responses were transcribed and analyzed using thematic analysis by identifying common themes and assigning codes as they appeared throughout interview transcriptions.

Participants

Survey responses were received from 74 parents of children with clubfoot, and 23 participated in the semi-structured interview with the research team. Demographic data of the study participants is presented in Table 1. Respondents primarily consisted of mothers who identified their race as White and hailed from various geographic areas, most predominantly in the United States and the United Kingdom. Over 75% of parent participants had attained an undergraduate education and had a median household income between $100,000 to $149,999 USD. Most had privately funded healthcare insurance and lived in households with a median of four total members. As summarized in Table 2, 62% of parents in the study had children diagnosed with bilateral clubfeet. Approximately 57% of parents reported that their child experienced a relapse of clubfoot deformity, which had occurred around an average age of 4.3 years. Almost all children underwent casting/bracing treatment and a significant proportion required surgery with an Achilles tenotomy and/or tendon transfer surgery.

**Table 1:** Demographics of study survey respondents IQR: interquartile range; SD: standard deviation

Variables			
Child age, years, mean [SD]		8.6	[3.5]
Child gender, n (%)	Female	21	(28%)
	Male	53	(72%)
Parent age, years, mean [SD]		40.0	[6.4]
Parent gender, n (%)	Female	69	(93%)
	Male	5	(7%)
Parent race, n (%)	White	61	(84%)
	Other	5	(7%)
	Black	3	(4%)
	Asian	2	(3%)
	Hispanic	2	(3%)
Parent marital status, n (%)	Married	60	(81%)
	Single (never married)	8	(11%)
	Single (divorced/separated)	3	(4%)
	Domestic partnership	3	(4%)
Parent employment status, n (%)	Full-time	34	(46%)
	Not employed	20	(27%)
	Part-time	18	(24%)
	Disabled	1	(1%)
	Student	1	(1%)
Parent education, n (%)	Postgraduate	31	(42%)
	Undergraduate	27	(36%)
	Some college	10	(14%)
	High school	6	(8%)
Parent insurance type, n (%)	Privately funded	45	(61%)
	Government-funded	16	(22%)
	None	13	(18%)
Household yearly income, n (%)	>$250,000	10	(14%)
	$200,000–$249,999	5	(7%)
	$150,000–$199,999	6	(8%)
	$100,000–$149,999	21	(28%)
	$50,000–$99,999	13	(18%)
	19	(26%)
Household size, median [IQR]		4	[4-5]

**Table 2:** Summary of clubfoot characteristics and treatment for children of the participants *Other treatments: ankle foot orthosis (AFO), psychological therapy, baby yoga, abduction dorsiflexion mechanism (ADM) brace. **Other surgical procedures: amputation, tibial derotational osteotomy, calcaneal osteotomy, plantar fasciotomy SD: standard deviation

Variables		N	(%)
Clubfoot type	Unilateral	28	(38%)
	Bilateral	46	(62%)
Treatment	Casting/bracing	73	(99%)
	Surgery	54	(73%)
	Physical therapy	43	(58%)
	Other*	7	(9%)
Surgical procedure	Achilles tenotomy	53	(72%)
	Tendon transfer	29	(39%)
	Other**	4	(5%)
Clubfoot relapse		42	(57%)
Age at first relapse, mean [SD]		4.3	[2.1]

## Results

Study questionnaire: discussions about clubfoot

**Figure 1:**
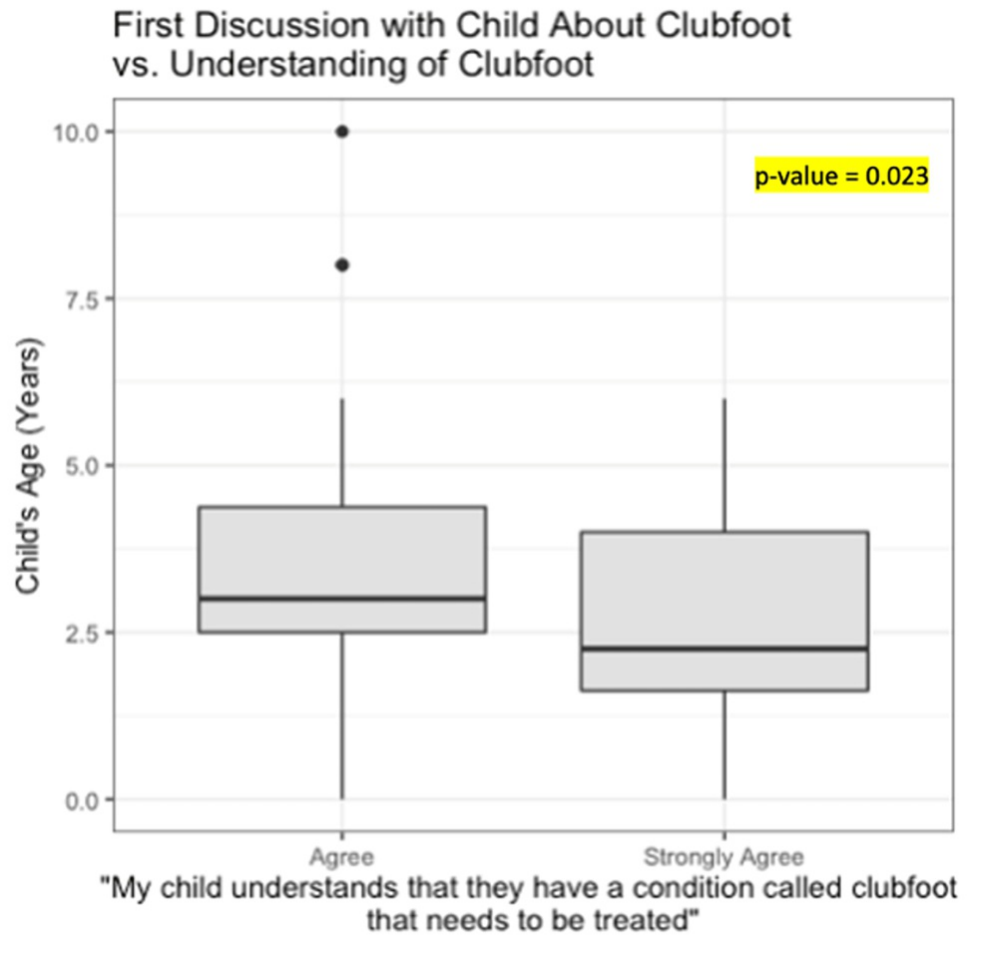
Children's level of understanding about clubfoot vs. age at which first discussion was initiated by family about their condition

Overall, 91% of parents reported having discussions about clubfoot with their children, beginning at a median age of three years (IQR: two to four years). The age at which parents first discussed clubfoot with their child was significantly lower for those who “strongly agree” that their child understands their condition versus those who “agree” (p=0.023) (Figure 1). When asked how they knew their child was ready to have conversations about clubfoot, parents noted initiating discussions upon their child commenting about differences between themselves and peers, and before or during various phases of treatment. For some families, clubfoot was always a topic of open discussion and they continued to share information with their child based on perceived ability of understanding and communication. The majority of respondents mentioned that they began speaking more openly about clubfoot with their children because they asked questions related to their condition and treatment (Table 3). The types of questions parents were asked by their children about clubfoot are listed in Table 3, with the top five most common topics being the following: past and future treatment, why they have clubfoot, why treatment like bracing and surgery is needed, the duration of clubfoot treatment, and whether clubfoot or its treatment causes pain. Additionally, children asked questions about why they need to undergo certain treatments that their siblings or peers do not require, and whether having clubfoot can affect their abilities in the future.

**Table 3:** Summary of discussions between parents and their children about clubfoot *Other resources used in preparation: children's books about clubfoot. **Other resources used when speaking to the child: photos before, during, and after clubfoot treatment. ***Other resources that would have been helpful: meeting with other clubfoot families

Discussion		N	(%)
Have discussed clubfoot with the child		67	(91%)
Child's age when first discussing clubfoot, years, median [IQR]		3	[2-4]
How did you know the child was ready to discuss clubfoot?	The child asked related questions	27	(40%)
	Clubfoot was always discussed	25	(37%)
	The child noticed differences from others	13	(19%)
	Discussed before/during treatment	10	(15%)
	Ability to communicate/understand	3	(4%)
Had concerns about the first clubfoot-related discussion		12	(16%)
What concerns did parents have?	Self-esteem	9	(75%)
	Child's anxiety	2	(17%)
	Not having all the answers	2	(17%)
	Child's response	2	(17%)
	Bringing up trauma/fear of doctors	2	(17%)
	Child's awareness about disability status	1	(8%)
	Child's pain awareness	1	(8%)
	The child won't want to continue the treatment	1	(8%)
The child asked parents questions about clubfoot		57	(77%)
What questions did your child ask?	What did/will treatment involve?	22	(39%)
	Why do I have clubfoot?	18	(32%)
	Why do I need surgery/bracing/treatment?	15	(26%)
	How long will I have clubfoot/need treatment?	10	(18%)
	Will I have pain? Is this why I feel pain?	9	(16%)
	Why am I not like my siblings/others?	5	(9%)
	Will this affect my abilities?	4	(7%)
	Age when treatment happened	2	(4%)
	What is clubfoot?	1	(2%)
	Will my children also have clubfoot?	1	(2%)
	Why don't others understand me?	1	(2%)
	What will my outcomes be in the future?	1	(2%)
Received guidance from the orthopedic provider for discussions		13	(18%)
Resources used to prepare to speak with the child about clubfoot	Online resources	38	(51%)
	Social media	36	(49%)
	Healthcare provider	26	(35%)
	Other parents of children with clubfoot	25	(34%)
	None	25	(34%)
	Printed Materials	14	(19%)
	Other*	5	(7%)
	Haven't discussed it yet	6	(8%)
Resources used when speaking to a child about clubfoot	None	41	(55%)
	Online resources	22	(30%)
	Other**	10	(14%)
	Children's books	8	(11%)
	Haven't discussed it yet	6	(8%)
Resources that would have been additionally helpful	Parent support group	54	(73%)
	Guidance from the healthcare provider	50	(68%)
	Online resources	49	(66%)
	Printed materials	33	(45%)
	Other***	3	(4%)

Prior to initiating conversations about clubfoot with their child, a subset of parents noted having certain concerns, mainly stemming from the possibility of the discussion affecting their child’s self-esteem. Other concerns parents had before sharing information about clubfoot included causing their child anxiety, being unsure of their child’s reaction, not having answers to their child’s questions, and further contributing to increased awareness about their child’s condition (Table 3).

**Figure 2:**
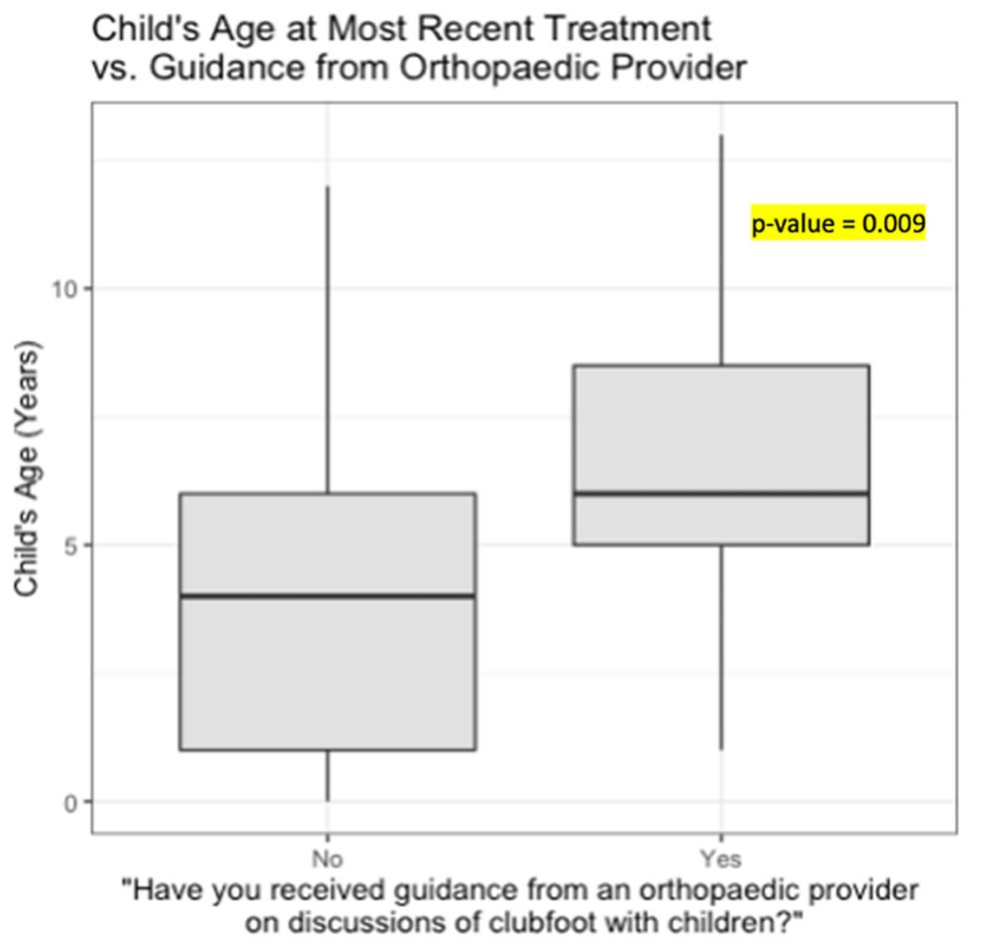
Child’s age at most recent treatment for clubfoot vs. response to whether the parent received guidance from the orthopedic provider about discussions surrounding the child's condition.

When asked about resources used in preparation for conversations about clubfoot with their child, the top four sources of information for parents included online web resources, social media, their child’s healthcare provider, and other families of children with clubfoot. While most parents indicated that they did not use any resources when sharing information about clubfoot with their child, some parents did mention using online web resources, showing photos before, during, and after casting and bracing treatment, and reading clubfoot-focused children’s books (Table 4). Although 68% of parents agreed that guidance from their orthopedic provider would have been helpful prior to engaging in conversations about clubfoot with their child, only 18% noted having interactions during which they were provided direct advice. Parents who received guidance from their provider on how to initiate these discussions had children who were receiving treatment at an older age than those who did not (p=0.009) (Figure 2). Apart from advice from an orthopedic provider, access to parent support groups was ranked the most important resource that would have been helpful for caregivers in preparing to speak to their child about their condition.

**Table 4:** Impact of living with clubfoot on child and the median age at which the child first expressed related concern IQR: interquartile range

		N	(%)	Median age, years	[IQR]
Concerns expressed by the child	Pain	44	(59%)	4	[3-5]
	Stiffness	29	(39%)	5.5	[3-8]
	Difficulty performing activities	35	(47%)	3	[3-6]
	Social interactions	22	(30%)	5	[5-6.5]
	Physical appearance of feet	19	(26%)	7	[4-8]
	Physical appearance of legs	13	(18%)	8	[5-10.5]

Study questionnaire: impact of living with clubfoot on children

The impact that living with clubfoot had on the child was ascertained from the parent's perspective across the following domains - pain, stiffness, difficulty performing activities, social interactions, and physical appearance of feet and legs (Table 4).

Pain

Approximately 59% of parents stated their child had expressed concerns with foot and ankle pain beginning at a median age of four years. Children noted pain after extended periods of physical activity, walking long distances, as a result of their Achilles tendon feeling tight, from pressure sores caused by brace wear, and occasionally experienced pain at nighttime.

Stiffness

Of note, 39% of parents recalled that their child experienced stiffness with the first mention of this concern around 5.5 years upon waking up in the morning, performing long periods of physical activity, and during gym class at school.

Difficulty Performing Activities

About 47% of parents noted their child had difficulty in performing certain activities such as walking long distances, attempting to run fast, standing on one leg, and kicking the ball when playing soccer. Other activities that were also occasionally challenging included riding a bike, ice-skating, and performing certain dancing, jumping, kicking, and gymnastics maneuvers. Children began commenting about these concerns at around a median age of three years.

Social Interactions

Of note, 30% of parents indicated that from a median age of five years, their child shared concerns about social interactions that were directly related to their clubfoot. These concerns included running slower while playing or taking part in physical activities when compared with their peers, feeling uncomfortable with wearing shorts that revealed a brace they may be wearing underneath, not feeling included at school as peers made comments about perceivable differences, and hearing direct remarks from others about wearing a cast or using a wheelchair during treatment for relapse. 

Physical Appearance of Feet/Legs

Approximately 26% of parents noted their child shared concerns about the physical appearance of their feet and 18% commented on their legs at around a median age of seven and eight years respectively. Children appreciated differences in the size of their feet, which appeared smaller in comparison to their peers and siblings or were two different sizes and impacted their ability to purchase footwear. They were also self-conscious about their calves, which were unequal in size with the affected side being substantially smaller in circumference, and surgical scars on their feet from clubfoot treatment. As a result, this was a subject of comparison between children and their peers, and they mentioned feeling like their leggings and pants felt different on either side.

Apart from the experiences shared above, parents also commented on the positive impact that living with clubfoot had on their children. Some parents described their children were “stronger”, “more resilient”, and “determined” on account of enduring their treatment journey. Others seemed to be more comfortable with physical differences and came to understand that people have different abilities and limitations of their own. A few parents also noted that their children learned to take responsibility for overcoming their condition as they grew. 

Semi-structured interview

**Figure 3:**
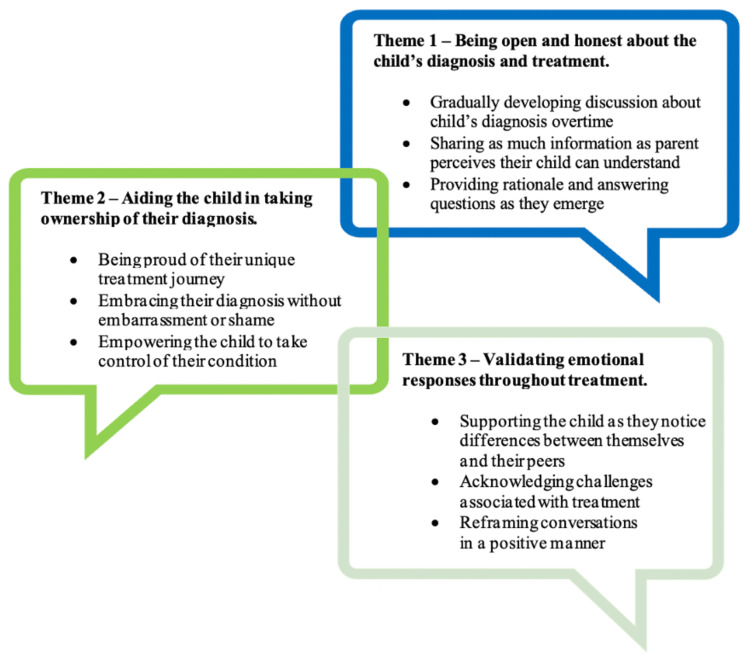
Major themes and subthemes identified during semi-structured interviews with parents

Three major themes that were identified along with associated subthemes across the semi-structured interviews are presented in Figure 3.

Theme 1: Being Open and Honest About the Child’s Diagnosis and Treatment

Subtheme 1a - gradually developing discussions about the child’s diagnosis over time: parents who had discussed clubfoot with their children shared that there was no “specific moment” or “perfect time” that they wanted to be open up about their diagnosis. They mentioned beginning discussions early, “always being open with what’s going on”, and continuing to “explain everything as they were going through it”. These discussions were described as “candid conversations” that were “fluid through time that evolved” throughout the child’s treatment. Parents also noted that discussion about their child’s clubfoot was a “necessary conversation for them to have” and was a topic that was revisited many times as the child continued to grow. Some families shared the importance of gradually introducing conversations about clubfoot to help prevent feelings of fear since “leaning into it is less scary if you provide little tidbits”. As one parent expressed, “nothing is worse than not knowing…when you don’t know, you can fear the unknown…but if you have the facts, you have the tools to deal with whatever your situation is.”

Subtheme 1b - sharing as much information as parent perceives their child can understand: sharing information that was “age appropriate” was important during conversations between parents and their children. Some stated that the “biggest concern was trying to make sure they explained it in terms” their child would be able to comprehend. While having these discussions or answering their child’s questions, parents noted the importance of balancing being “forward”, “open”, “honest”, and “consistent” while also thinking about “whether that information is something that they can understand at their age”. These conversations were helpful in normalizing the ongoing discussions surrounding clubfoot and gradually allowed the child to learn more about their condition.

Subtheme 1c - providing rationale and answering questions as they emerged: being able to answer a child’s questions about their condition and share the reasoning behind the importance of treatment provided opportunities for parents to educate their child about clubfoot. Parents answered questions about why their child has clubfoot by stating that “you were born with your foot in a certain way, that’s why you’re wearing these boots in the night” and “this is why we go see the doctor”. They mentioned that explaining things like the purpose of bracing, the need for various appointments, and why stretching and physical therapy were part of their individual routine helped their child understand that clubfoot “is something that is particular to them, and it needed to be treated”. Many families noted challenges with their child resisting wearing the brace as they continued to grow. During these moments, parents recalled that “the more that we could teach, the less of a fight there was or resistance with the treatment”. Some explanations parents shared with their children to help them learn about their treatments involved statements related to performing activities and the positioning of their foot, including “to keep your feet healthy”, “to keep your feet straight”, “to get to a correct position”, and “to walk and run, continue to jump and be a fast runner”. Answering openly and honestly “whenever any little questions came up” and providing a rationale for their child’s needs allowed them to “listen better” and continue to develop their understanding of clubfoot.

Theme 2: Aiding Children in Taking Ownership of Their Diagnosis

Subtheme 2a - being proud of their unique treatment journey: parents mentioned that helping their child “be in touch with what they went through”, including early treatments they do not remember, allowed their child to “be proud of how far they have come and not to forget”. Sharing pictures and clubfoot casts saved from initial treatment days allowed parents to show their children the progress in their treatment. Parents shared sentiments like “we wanted her to understand so that she can understand and explain it to others”, “she takes huge pride in what her feet looked like when she was a baby and what she’s accomplished”, and “she’s so proud of her feet, what she’s done, and she shows people her scars and everything”. Several parents stated that guiding their children to be proud of their clubfoot helped them accept that it is “part of who I am” and enabled them to speak openly about their condition. Their children were even able to “explain it to friends” and were “confident to tell them why” they would wear the brace when around family or peers at gatherings, sleepovers, or school.

Subtheme 2b - embracing their diagnosis without embarrassment or shame: along with expressing the desire to help their children be proud of their condition, parents emphasized the importance of ensuring their children would “never be ashamed or embarrassed” because of their clubfoot. One parent stated, “I wanted him to have good feelings when he thought about his feet and not be embarrassed or it be a subject we don’t talk about”. Normalizing discussions about clubfoot and related treatment helped parents show their children that “this is a whole family thing” and that “we were going to help”. Another parent shared that their family decided not to “hide it from people”. “We weren’t going to play the 'all is well with us' card, or that we had a child that was flawed - that was not how we were going to look at this; instead, we were going to look at this as our child has this thing that we’re going to overcome”.

Subtheme 2c - empowering children to take control of their condition: one of the goals expressed by parents in educating their children about clubfoot involved empowering their children with the support and information they needed to “take ownership and take control” of their condition. As summarized by one parent, informing their child about their clubfoot and treatment “is to genuinely help her have the choice to live the life that she wants to live…she needs to be aware of her condition, she needs to be aware of what treatments have been done to her, and one or two options moving forward.” Sharing information openly about clubfoot allows the child to take responsibility for their body and health, and ultimately “give them as much agency as we can”.

Theme 3: Validating Emotional Responses Throughout Treatment

Subtheme 3a - supporting the child as they notice differences between themselves and their peers: parents noted that their children were observant from a young age and “started realizing that maybe I’m doing something a little bit different than my friends or my peers”. Once they were able to “socialize with other kids and compare that maybe their routine was different”, their curiosity and questioning continued to peak. This led the children to ask parents questions about their clubfoot and treatment after noticing differences between themselves, siblings, and peers. Children would ask why they had to do physical therapy, wear a cast or brace, and why they couldn’t do “things the other children were doing” upon “realizing that not everybody had casts and braces”. They also questioned why they were “taking a day off” and “traveling for treatment”. As the children grew, parents recalled them “getting quite frustrated”, and feeling “down” and “upset” when comparing themselves to peers who would be able to run faster, perform activities for longer periods of time without their feet tiring, or would be wearing “big kid sneakers” earlier than they would be able to fit into them. Parents felt that “obviously we needed to address it” because of the impact this has on their children and “it does affect them socially”, especially when peers or siblings were “quite verbal” and “managed to point out differences”.

Subtheme 3b - acknowledging challenges associated with treatment: although clubfoot treatment follows a similar trajectory of progression for most children, parents acknowledged that “everyone’s journey and processes are different”. A sentiment shared by several families was that “the concerns never go away…they just get different” as their child continued through stages of treatment. “Those moments where the emotions come into it” can be especially challenging for children as they navigate these experiences and for parents as they support them through it. As one parent noted, “I think our responsibility as parents is to not shy away from those difficult conversations, because you are the best person to know when the right time to have those conversations is”. Acknowledging concerns and challenges associated with their children’s treatment journey as they arise allows parents to maintain open communication and normalize conversations about clubfoot.

Subtheme 3c - reframing conversations in a positive manner: parents believed that their children mirrored the attitudes with which they approached conversations about clubfoot. This made it important for them to answer questions honestly and in a positive manner. When referring to the tone their family adapts for discussions surrounding their child’s clubfoot, one parent expressed that “when you project the positive, go-getter perseverance attitude, that’s how they’re going to be”. By taking a positive approach to their conversations, families felt they were able to help their children become more “accepting” of their condition. This also helped reinforce to their child that their clubfoot would not limit their functioning substantially and to instead be proud of their progress.

## Discussion

The experience of disclosing a medical diagnosis to a patient can be challenging and it becomes even more difficult when parents need to educate their children about a congenital condition that requires significant treatment. As the children grow, they become more attuned to their surroundings, leading them to take note and question differences in appearance, behaviors, and routines. For parents, balancing aspects like sharing accurate information, handling emotional responses, and normalizing their children’s lived experiences becomes part of an ongoing process to support the children in taking ownership of their condition [9].

Research investigating interactions that take place between parents and their children surrounding the disclosure of a diagnosis is extremely limited, and the few available studies focus on autism spectrum disorder and pediatric cancer [8,9]. A common theme noted across available literature and in our study findings was the importance of being open and honest when conveying information to children about their condition. Based on our experiences shared during the semi-structured interviews, parents highlighted this theme along with how gradually developing discussions over time, sharing information based on their child’s level of understanding, and providing a rationale to answer their child’s questions helped to maintain open communication about clubfoot. By encouraging the children to embrace their diagnosis, take control of their condition, and be proud of their treatment journey, parents were able to guide their children to slowly take ownership of their health. Also, acknowledging the challenges of clubfoot treatment and adopting a positive mindset to overcome them allowed parents to support their children through various phases of treatment.

A common concern that parents had prior to sharing information with their children about their diagnosis included causing emotional distress or affecting their self-esteem - a factor that could potentially affect how and when parents begin these critical discussions. A study by Last and Van Veldhuizen described that children who seemed to understand their cancer diagnosis from an early age experienced less anxiety and depression compared to those who received incomplete information about their condition [11]. Our study found that the age at which parents initially discussed clubfoot with their child was significantly lower for those who “strongly agree” that their child understood their condition compared to those who “agree”. This implies that early discussions with children about their medical diagnosis may not only allow the children to better manage emotional responses but also contribute to a deeper understanding of their condition.

In an effort to encourage families to have open conversations about clubfoot, it is also important to assess ways to support them regarding this exchange. Among our study participants, access to parent support groups was ranked the most important resource to prepare for discussions with children about their clubfoot diagnosis. Parent-to-parent social support can supply valuable information through shared experiences and resources. This form of near-peer support also provides emotional empowerment to help caregivers process information necessary to aid their children [12,13]. In addition to the support from families who have undergone similar experiences, 68% of parents in our study agreed that guidance from their child’s orthopedic provider would be the next most helpful resource. While parents expressed that the support of healthcare professionals would be beneficial, only 18% of study participants noted receiving focused advice about how to speak to their child about clubfoot. Addressing aspects of caring for a child beyond pure medical treatment, including how to disclose a diagnosis and support the child in learning about their condition, presents a critical opportunity to provide care that is patient-centric and comprehensive. This also enables the provider to develop an enduring relationship with families, which is sensitive to their unique needs and instills greater confidence in the caregiver’s abilities to support their children across multiple facets of their treatment journey [14,15].

The acknowledgment and understanding of how an individual’s health affects various domains of their life can uncover additional areas for targeted support. Recent evidence from semi-structured interviews among children affected with lower limb deformities reveals five key factors that mediate health-related quality of life: appearance, physical health, psychological health, school, and social health [16]. When parent participants in our study were asked to recall concerns voiced by their child about pain, stiffness, difficulty performing activities, social interactions, and physical appearance that were directly related to living with clubfoot, the experiences and comments that were shared also encompassed similar spheres. Awareness about the influence a structural diagnosis has across these domains can educate parents about realistic expectations learned from the collective experience and how they may evolve as the child grows. Parents and providers may also use these overarching themes to create a conceptual framework from which they can answer questions and engage in meaningful discussions to gradually guide the children in learning about their diagnosis over time.

While this study provides an in-depth look into how parents navigate conversations with their children about a physical diagnosis, there are also certain limitations to be noted. The study questionnaire did not use validated surveys and was developed by our research team from a review of literature and collective experience across several disciplines. The questionnaire was pilot-tested with a group of clubfoot families and suggestions were incorporated prior to beginning data collection. Responses to the questionnaire are also subject to recall bias as parents answered questions based on past interactions and experiences. As study recruitment occurred through clubfoot support groups on Facebook and captured the experiences of a subset of parents who are actively engaged in these online forums, there is potential for sampling bias as the findings may not be generalizable to the overall clubfoot community. We also recognize that findings may vary based on regional availability and accessibility of resources and information. Finally, while the study may provide valuable advice that can be helpful for parents of children affected by a range of physical conditions, our study specifically looks at the experiences of clubfoot children and families, which may affect the applicability of the results.

The findings of this study along with recurrent themes from conversations with clubfoot families provide helpful strategies to aid children in learning and taking ownership of their condition. As an extension of the study efforts, further investigation examining how direct provider-parent counseling on disclosing a medical diagnosis may affect caregiver confidence in facilitating these discussions may be useful. In addition to parent and provider guidance, evaluating the effect of peer-to-peer support among children affected by structural conditions on knowledge of their health may also help devise constructive ways to support them in this process.

## Conclusions

Disclosure of a medical diagnosis can be especially challenging for parents who may need to educate their children about a condition that requires significant treatment beginning early in life. As revealed in this study, which provides insights into initiating conversations with children about congenital structural diagnoses, the implications for parents and clinicians may be substantial. By exploring discussions among clubfoot families and understanding how parents navigate these critical conversations, several themes have been uncovered, which may also be applicable to caregivers helping children learn more about their diagnosis as they grow. This study also uncovers the significance that near-peer advice from families who have undergone similar experiences and direct advice from healthcare providers in speaking to children about their diagnosis can have in aiding families to support their children beyond purely physical treatment.
